# Determinants of uncontrolled asthma in a Swedish asthma population: cross-sectional observational study

**DOI:** 10.3402/ecrj.v1.24109

**Published:** 2014-09-12

**Authors:** Mary Kämpe, Karin Lisspers, Björn Ställberg, Josefin Sundh, Scott Montgomery, Christer Janson

**Affiliations:** 1 Department of Medical Sciences, Respiratory Medicine and Allergology, Uppsala University Uppsala, Sweden; 2 Department of Public Health and Caring Science, Family Medicine and Preventive Medicine, Uppsala University, Uppsala, Sweden; 3 Department of Respiratory Medicine, Örebro University Hospital, Örebro, Sweden; 4 Clinical Epidemiology and Biostatistics, Örebro University Hospital and Örebro University, Örebro, Sweden; 5 Clinical Epidemiology Unit, Department of Medicine, Karolinska University Hospital, Karolinska Institute, Stockholm, Sweden; 6 Department of Epidemiology and Public Health, University College London, UK

**Keywords:** asthma, uncontrolled, risk factors, quality of life, cross-sectional study

## Abstract

**Background:**

Asthma control is achieved in a low proportion of patients. The primary aim was to evaluate risk factors for uncontrolled asthma. The secondary aim was to assess quality of life associated with asthma control.

**Methods:**

In a cross-sectional study, asthma patients aged 18–75 were randomly selected from primary and secondary health care centers. Postal questionnaires were sent to 1,675 patients and the response rate was 71%. A total of 846 patients from primary and 341 patients from secondary care were evaluated. Data were collected using a questionnaire and review of medical records. The questionnaire included questions about asthma control and a quality-of-life questionnaire, the mini-AQLQ, with four domains (symptoms, activity limitation, emotional function, and environmental stimuli). The mean score for each domain and the overall score were calculated. Asthma control was divided into three levels according to the GINA guidelines and partly and uncontrolled asthma were combined into one group – poorly controlled asthma.

**Results:**

Asthma control was achieved in 36% of the sample: 38% in primary and 29% in secondary care. In primary and secondary care, 35 and 45% had uncontrolled asthma, respectively. Risk factors for poorly controlled asthma were female sex [OR 1.31 (1.003–1.70)], older age [OR 2.18 (1.28–3.73)], lower educational level [OR 1.63 (1.14–2.33)], and current smoking [OR 1.68 (1.16–2.43)]. Older age and lower educational level remained statistically significantly associated with poorly controlled asthma when the analyses were limited to never-smokers. Depression was an independent risk factor for poorly controlled asthma in men [OR 3.44 (1.12–10.54)]. The mini-AQLQ scores and the mean overall score were significantly lower in uncontrolled asthma.

**Conclusion:**

Risk factors for poorly controlled asthma were female sex, older age, low educational level, and smoking. Uncontrolled asthma was significantly associated with lower quality of life.

Asthma is a chronic disease characterized by airway inflammation, variable airflow obstruction, and hyperresponsiveness. In some patients, remodeling leads to a decline in lung function. Impaired quality of life is common among asthmatics, especially if their disease is not treated effectively. The disease is widespread, globally affecting more than 300 million people, and generating a substantial socioeconomic burden for patients and society (1).

The main goal for asthma management is to achieve and maintain asthma control (2). Asthma control is needed on both a day-to-day basis as well as to minimize future risks of exacerbations and decline in lung function (2, 3). The shortcomings in asthma treatment have been demonstrated repeatedly in observational studies over time and this has resulted in switching from severity to control-based guidelines for asthma management in the updated GINA guidelines from 2006 (2) and the US guidelines from 2007 (4). In spite of new guidelines, asthma control is still not accomplished in the majority of patients according to population studies during the past decade, (5–7) even though the vast majority of asthma is mild to moderate and should respond to modern therapeutic regimens (8).

Poorly controlled asthma affects the patient's daily life with an impact on both mental and physical health and is associated with lower professional achievement (9) and socioeconomic disadvantage (1). Hence, it is of importance to achieve adequate asthma control and explore reasons for difficult-to-control asthma as well as identifying risk factors for poor asthma control. Patient education, action plans, and knowledge of self-management is crucial for an optimal outcome over time and future risk reduction. In addition, periodic clinical evaluation is necessary to assess adherence to the management plan (10).

The present study was part of a multicenter patient survey in central Sweden investigating a cohort of asthma patients from both primary and secondary healthcare settings (7, 11). The primary aim of this study was to evaluate risk factors for poorly controlled asthma in a general Swedish population. The secondary aim was to assess quality of life associated with asthma control.

## Materials and methods

### Procedure and sampling

The study was conducted during the years 2000–2005 in central Sweden in an area including both rural and urban dwellings (7, 11) with approximately 1.9 million residents in seven counties. From each county, eight primary care centers and two hospitals were randomly selected: one with a department of respiratory medicine and one with a department of internal medicine from each county. In total, 56 primary and 14 secondary care centers were sampled.

**Fig. 1:**
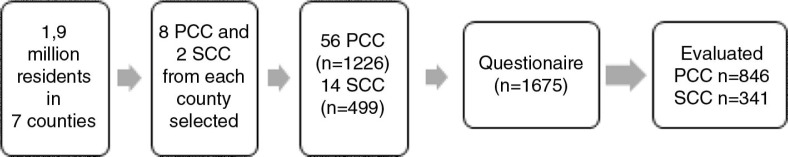
Flow chart for study conductance. Patients were selected from seven counties in central Sweden with approximately 1.9 million residents. (PCC=primary care center, SCC=secondary care center).

Each center generated a list of all patients with an asthma diagnosis (ICD-10 J45) who had attended the center during the past 4 years in the age range 18–75. From the lists in primary care centers, 22 patients were randomly selected, and from each list in secondary care 35 patients were randomly selected. If any center had fewer patients, all available patients were included. A total of 1,226 patients were included from the primary care centers and 499 patients from the hospitals (Fig. 1).

### Data collection

Data were collected in 2005 from self-completed questionnaires and review of medical records for the period of 2000–2003 (inclusive). The response rate for the postal questionnaire was 71 and 98% of these respondents consenting to a review of their medical records. Complete information for all analyzed variables was available for 1,187 patients: 846 patients from primary care and 341 patients from secondary care. Two research nurses entered data from the questionnaires and medical records (Fig. 1).

### Patient characteristics

Information on sex, age, body mass index (BMI), smoking history, level of education, current medication and symptoms, as well as emergency consultations was collected from the questionnaires. Data on procedures for diagnosis and history of allergy were obtained from the medical records, as was information about co-morbid disease. Smokers were divided into daily smokers and ex/occasional smokers, where occasional smokers had a history of intermittent smoking. Depression was defined as having a diagnosis of depression in combination with antidepressant drug treatment.

### Asthma control

The definition of asthma control was divided into three levels according to GINA guidelines from 2006, that is, controlled, partly controlled, and uncontrolled asthma (2) (Table 1). The GINA classification was slightly modified in the sense that lung function was not used. Classification of asthma control in the study was based on two questions concerning the previous week: use of ≤two doses of short-acting β_2_-agonists and no night awakenings and two questions concerning the past 6 months (no emergency visits or oral courses of steroids). An asthma exacerbation was defined as an emergency visit or a course of oral steroids. Uncontrolled asthma had at least one exacerbation during the past 6 months (7, 11). Partly controlled and uncontrolled asthma were combined as poor asthma control in some calculations.

**Table 1:** Criteria for asthma control based on GINA guidelines 2006, with the exception of lung function, which was not used in the present classification

	Controlled asthma	Partly controlled asthma		Uncontrolled asthma^a^	
**Night awakenings in the past week**	None	≥1 time	*Either* of these	≥1 time	*Both* of these^a^
**Need for reliever in the past week because of asthma symptoms**	≤2 times	≥3 times		≥3 times	
**Exacerbations in the past 6 months;** unscheduled visits *or* oral course of steroids	None	None		≥1 time^a^	

a Definition of uncontrolled asthma: either exacerbation or night awakenings and need for reliever.

### Quality of life

The questionnaire included a validated asthma-specific quality-of-life questionnaire, the mini-AQLQ (12), where patients were asked to assess functional impairment due to their asthma. Patients were requested to recall their experiences during the past 2 weeks and respond to 15 questions grouped into four domains on a seven-point scale. The four domains are: symptoms (five items), activity limitations (four items), emotional function (three items), and environmental stimuli (three items). Each question was scored from severe impairment (1) to no impairment (7) and the mean score for each of the four domains, as well as the overall score, was calculated.

### Statistical analysis

Descriptive statistics were used standard parametric methods. Differences between groups for continuous data were tested with the Student's *t*-test or analyses of variance. Differences in proportions were assessed using the Chi-square test. Multiple logistic regression was used in the multivariate analyses. In these analyses patients with partly and uncontrolled asthma were combined into one group – poorly controlled asthma. Independent variables that were statistically significantly (*p*<0.05) associated with asthma control in the univariate analyses were included in the multivariate analyses. Stratified analyses were performed for men and women and for never- and ever-smokers. Interaction analyses were performed for effect modification by sex and smoking history.

### Ethics

The study was performed in accordance with the Declaration of Helsinki and with the approval of the Ethics Committee at the Medical Faculty at Uppsala University (DNR 2004: M-445). Patients were included in the study only after informed written consent was obtained.

## Results

### General characteristics

Baseline demographic and clinical characteristics are presented in Table 2. Asthma control was only achieved in 36% of the patients in the study. In univariate analyses, uncontrolled asthma was associated with female sex, older age, higher BMI, and current smoking. There was a trend toward more frequent comorbidity such as heart disease, hypertension, and diabetes in the patient group with uncontrolled and partly controlled asthma, that is, poorly controlled asthma.

**Table 2:** Baseline characteristics of the overall population sample for controlled, partly controlled, and uncontrolled asthma (absolute number and percentage in brackets)

	Controlled asthma (*n* and %)	Partly controlled asthma (*n* and %)	Uncontrolled asthma (*n* and %)	*p*
Total study group				
*n*=1,187	*n*=422 (36%)	*n*=313 (26%)	*n*=452 (38%)	
Sex				
Female	238 (56.4)	184 (58.8)	292 (64.6)	0.04
Age group				
18–29 years	70 (16.6)	44 (14.2)	43 (9.6)	0.01
30–49 years	139 (33.0)	88 (28.4)	135 (30.2)	
50–64 years	148 (35.2)	111 (35.8)	167 (37.4)	
≥65 years	64 (15.2)	67 (21.6)	102 (22.9)	
Body mass index (kg×m^2^)				
<20	18 (4.4)	7 (2.3)	19 (4.4)	0.03
20–<25	159 (38.5)	117 (38.1)	134 (30.8)	
25–<30	155 (37.5)	124 (40.4)	166 (38.2)	
≥30	81 (19.6)	59 (19.2)	116 (26.7)	
Education				
Primary school (≤9 years)	112 (27.1)	114 (37.5)	174 (39.8)	<0.001
Secondary school (11 years)	96 (23.2)	63 (20.7)	107 (24.5)	
Further education (12 years)	76 (18.4)	59 (19.4)	64 (14.6)	
Higher education	129 (31.2)	68 (22.4)	92 (20.0)	
Smoking group				
Never smoker	248 (59.0)	172 (55.0)	205 (45.6)	<0.001
Ex/occasional smoker	115 (27.4)	91 (29.1)	147 (32.7)	
Current smoker	57 (13.6)	50 (16.0)	98 (21.8)	
Allergic rhinitis	125 (30.0)	106 (34.2)	120 (26.9)	0.10
Depression	29 (7.0)	27 (8.7)	51 (11.4)	0.07
Comorbidity				
Heart disease	32 (7.7)	33 (10.6)	51 (11.4)	0.16
Hypertension	62 (14.9)	59 (19.0)	74 (16.6)	0.34
Diabetes	24 (5.8)	23 (7.4)	31 (7.0)	0.64

### Level of health care

**Fig. 2:**
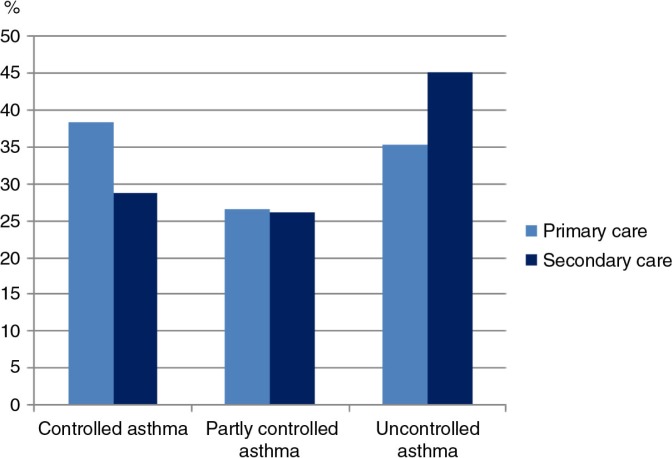
Asthma control in primary and secondary care (<0.0001).

Asthma control in primary care was achieved for 38% and in secondary care for 29% of the patients. In addition, in primary and secondary care, 35% compared with 45% had uncontrolled asthma, respectively (Fig. 2).

### Quality of life

The mini-AQLQ scores were significantly lower overall and in all four domains (symptoms, activity limitations, emotional function, and environmental stimuli) in uncontrolled compared with controlled asthma (Table 3). These differences remained statistically significant (*p*<0.001) after adjustment for age, sex, educational level, smoking, BMI, depression, and allergic rhinitis.

**Table 3:** Assessment of mini-AQLQ among those with controlled, partly controlled, and uncontrolled asthma for the past 2 weeks

	Controlled asthma	Partly controlled asthma	Uncontrolled asthma	*p*
AQLQ score (mean±SD)	6.15±0.78	5.27±1.13	4.54±1.21	<0.0001
Symptom score (mean±SD)	6.05±0.85	5.05±1.22	4.30±1.31	<0.0001
Activity limitation score (mean±SD)	6.32±0.88	5.57±1.28	4.89±1.40	<0.0001
Emotional function score (mean±SD)	6.25±0.97	5.32±1.42	4.46±1.57	<0.0001
Environmental stimuli score (mean±SD)	6.03±1.05	5.26±1.40	4.58±1.49	<0.0001

Scoring from 1 to 7, with no impairment for score 7.

### Treatment

**Fig. 3:**
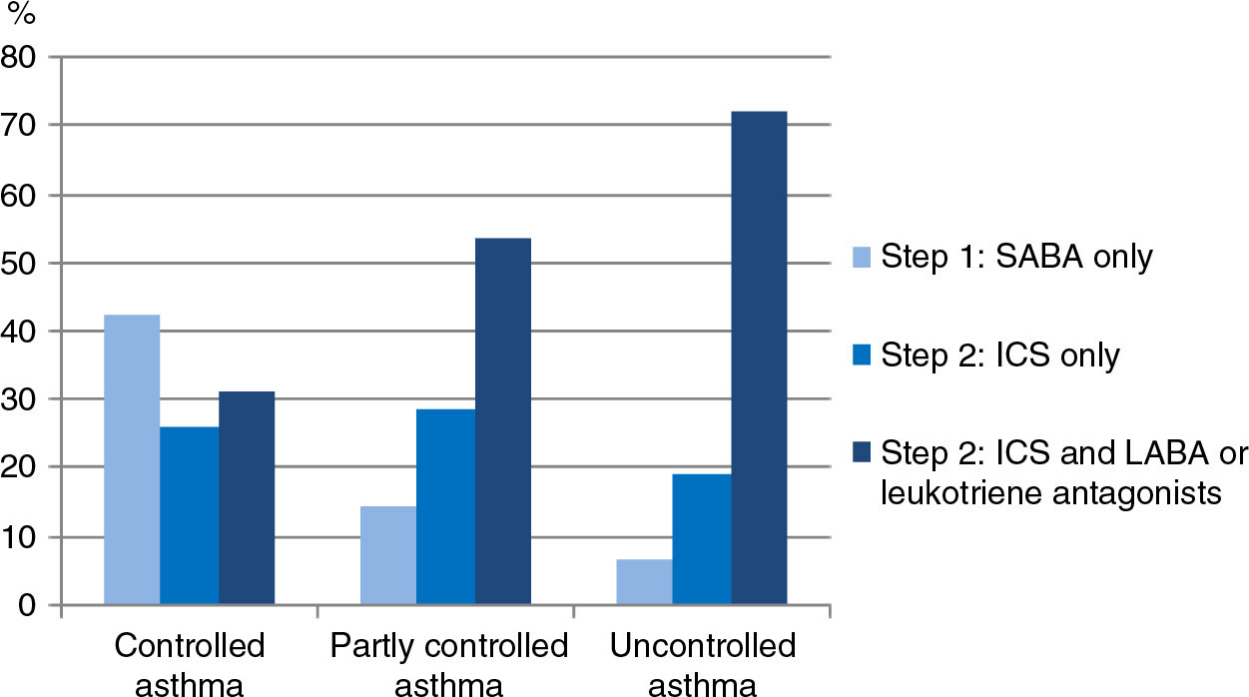
Asthma treatment steps. Step 1: only SABA on-demand. Step 2: ICS alone for maintenance and SABA on-demand. Step 3: ICS and LABA or leukotriene antagonists for maintenance and SABA on-demand.

In patients with controlled asthma, just over 40% were in receipt of rescue medication with short-acting β-agonist (SABA) alone and 58% received inhaled corticosteroids (ICS) alone or ICS in combination with long acting β-agonist (LABA) or leukotriene receptor agonists. In patients with uncontrolled asthma, 71% had regular treatment with ICS in combination with LABA or leukotriene receptor agonists and 19% received ICS as monotherapy for maintenance (Fig. 3).

### Risk factors for poorly controlled asthma

Independent risk factors for poorly controlled asthma in the overall population were female sex, older age (≥65 years), lower educational level, and current smoking when analyzed using multiple logistic regression (Table 4). In the age group ≥65 years, 44% had uncontrolled asthma and 29% had partly controlled asthma, that is, 73% had poor asthma control (Table 2).

**Table 4:** Odds ratio for not having controlled asthma adjusted for all the variables in the table

	Poorly controlled asthma Odds ratio (95% confidence interval) (*n*=786)
Sex	
Female	1.31 (1.00–1.70)
Age group	
<30	1
30–49 years	1.32 (0.86–2.04)
50–64 years	1.50 (0.96–2.36)
≥65 years	2.18 (1.28–3.73)
Educational level	
Higher education	1
Further education (12 years)	1.53 (1.02–2.29)
Secondary school (11 years)	1.35 (0.94–1.95)
Primary school (≤9 years)	1.63 (1.14–2.33)
BMI group (kg×m^2^)	
<20	0.90 (0.45–1.79)
20–<25	1
25–<30	1.14 (0.85–1.54)
≥30	1.20 (0.84–1.69)
Smoking group	
Never (*n*=377)	1
Ex/occasional smoker (*n*=238)	1.19 (0.88–1.61)
Current smoker (*n*=148)	1.68 (1.16–2.43)
Allergic rhinitis	1.26 (0.94–1.67)
Depression	1.39 (0.87–2.22)

Partly controlled and uncontrolled asthma combined to poorly controlled asthma in the calculations.

When men and women were analyzed separately, depression was independently associated with poorly controlled asthma in men [OR 3.44 (1.12–10.5)] but not in women [1.10 (0.64–1.87)]. This potential effect modification is not statistically significant when assessed using interaction testing (*p*_interaction_=0.07). Being obese (BMI>30 kg/m^2^) was also statistically significantly associated with not having controlled asthma in men but not in women OR 1.83 (1.01–3.32) and 0.98 (0.63–1.52), respectively. This difference was, however, not statistically significant when assessed using interaction testing (*p*_interaction_=0.11).

Older age and lower educational level remained significantly associated with poorly controlled asthma when the analysis was limited to never-smokers (Table 5). Allergic rhinitis was also related to a higher risk of having poorly controlled asthma in the never-smoker group. This interaction between smoking and allergic rhinitis is statistically significant (*p*_interaction_=0.03).

**Table 5:** Risk factors for having partly or uncontrolled asthma

	Never-smokers Odds ratio (95% confidence interval) (*n*=377)	Ever-smokers Odds ratio (95% confidence interval) (*n*=386)
Age group		
<30 years	1.34 (0.78–2.32)	1.04 (0.49–2.18)
30–49 years	1.85 (1.03–3.33)	0.92 (0.44–1.90)
50–64 years	2.38 (1.18–4.76)	1.46 (0.63–3.41)
≥65 years	1	1
Educational level		
Higher education	1	1
Further education (12 years)	1.82 (1.10–3.00)	1.54 (0.92–2.58)
Secondary school (11 years)	1.28 (0.79–2.07)	1.46 (0.84–2.55)
Primary school (≤9 years)	1.55 (0.94–2.58)	1.46 (0.75–2.86)
BMI group (kg×m^−2^)		
<20	0.96 (0.38–2.40)	0.91 (0.32–2.50)
20–<25	1	1
25–<30	1.46 (0.98–2.18)	0.71 (0.45–1.11)
≥30	1.39 (0.86–2.25)	0.89 (0.53–1.48)
Allergic rhinitis	1.72 (1.18–2.51)	0.80 (0.52–1.24)
Depression	1.48 (0.74–2.96)	1.51 (0.81–2.83)

Logistic regression stratified by smoking history.

## Discussion

The main finding of this observational study was that achieving asthma control is an ongoing challenge. In this cross-sectional survey from central Sweden, just over one-third of the patients had controlled asthma. The main risk factors for having partly or uncontrolled asthma were older age, female sex, low educational level, and smoking. In addition, uncontrolled asthma was significantly associated with a lower quality of life measured by the mini-AQLQ.

The low proportion of patients with controlled asthma is consistent with previous observational studies reporting lack of asthma control in the past decade. (5–7). In this general population study, asthma control was slightly higher in primary than secondary care. This is probably related to a selection of more severe asthmatics in secondary care. This data is in accordance with previous studies on asthma control. In the epidemiological study on the genetics and environment of asthma, bronchial hyperresponsiveness and atopy (EGEA 2), 26% had uncontrolled asthma and 30% partly controlled asthma (13); and in the European Community Respiratory Health Survey II (ECRHS II), 49% of ICS users had uncontrolled and 36% had partly controlled asthma, with only 15% achieving asthma control (5).

In this investigation, uncontrolled asthma was more common in the older age groups, as many as 73% had poor asthma control. Asthma among the elderly is of special concern as many patients have both concomitant diseases and polypharmacy with an increased risk of reduced treatment adherence (14). Aging is also associated with a decline in immune defenses, that is, innate and adaptive immunosenescence (15), as well as impaired mucociliary clearance (16). Consequently, elderly patients are at greater risk of infectious exacerbations. In addition, elderly people might also have a longer duration of asthma disease and hence a more pronounced reduction in lung function. In addition, they might have impaired cognitive capacity and consequently poor inhaler technique (17). Elderly people might also have lower expectations about the degree of control that is possible (14, 18). Interestingly, it has also been reported that the response to β-receptor agonists declines with age due to a decreased number of β_2_-receptors in smooth muscle (19, 20). Therefore, asthma in the older adults is a serious health issue as the prevalence of asthma in the elderly population is steadily increasing because of an increase in asthma incidence in the population together with a longer average life expectancy (21).

In this observational study, uncontrolled asthma was more common in women than men. These data are consistent with reports from other authors showing that women have both a higher incidence of adult onset asthma (22) as well as a higher frequency of poorly controlled asthma (23). There are speculations concerning whether females are more likely to perceive or acknowledge symptoms, (11, 24) though the differences may in part be specifically phenotype and hormone related (25). Conversely we found that BMI>30 kg×m^2^ and depression were risk factors for poor asthma control in men, but not in women.

A lower level of education was a risk factor for uncontrolled asthma, which is consistent with previous epidemiological studies (26). A higher level of education may facilitate adherence to treatment but the group with a higher educational level also is less likely to work in occupations with a high level of exposure to allergens and triggers (27, 28).

In patients with uncontrolled asthma, as many as 22% were current smokers compared with 14% in controlled asthma. Our results are consistent with other reports of smoking among asthma patients, who often have more severe disease than non-smokers (6). Particularly high rates of smoking have been reported in asthma patients who have frequent emergency visits (29), accelerated reduction in lung function (30), and an impaired therapeutic response to corticosteroids (31). Thus, asthma control in smokers is more difficult to achieve and this may partly be due to altered airway inflammation with lower nitric oxygen levels, less eosinophil (32), more neutrophil airway inflammation (33), and reduced lung function. In addition, smoking is a risk factor for developing asthma, especially in women (34). It should be noted that the associations of poorly controlled asthma with older age and low educational level remained statistically significant even when ex/occasional and current smokers were excluded from the analysis.

In this study, significant associations between high BMI and uncontrolled asthma could only be observed in men. However, other authors have previously reported a higher prevalence of asthma in obese compared with lean individuals in numerous cross-sectional studies. In addition, several prospective studies both in children and adults indicate that obesity pre-dates asthma, and that the relative risk of incident asthma increases with BMI (10). Obesity also appears to worsen asthma control (35) and some, but not all studies, indicate that it can increase asthma severity (36).

Depression, as well as anxiety, has been reported as risk factors for poor asthma control (37). In our study, depression was more common in women than men (data not shown) but depression was only related to poorly controlled asthma in men. Allergic rhinitis is similarly known as a risk factor for poorer asthma control and more severe asthma (25, 38); however, this could only be demonstrated in never-smokers in our study population. No significant association with asthma control was found between other comorbidities such as heart disease, hypertension, and diabetes in the present study.

As expected we found a clear association between the level of asthma control and asthma-specific quality of life. This result is in accordance with what has been found by other authors (10, 39). The mini-AQLQ scores in uncontrolled asthma were significantly lower for all four domains, (symptoms, activity limitations, emotional function, and environmental stimuli) as well as for the overall score. Previous studies have demonstrated that the mini-AQLQ instrument is valid and useful for monitoring quality of life in patients with asthma (40) and is also sensitive to asthma severity in population-based settings (41).

In this study, 71% of patients with uncontrolled asthma had regular treatment with ICS and LABA or leukotriene antagonists and 19% received ICS as monotherapy for maintenance according to those who provided answers in the questionnaire (approximately 90% of uncontrolled asthma patients received reasonably effective medication according to GINA guidelines). It has been argued that under-treatment and particularly under-usage of ICS is due to insufficient asthma control (42) but our results did not support this view. Generally, there are high standards of care available in both primary and secondary care in Sweden as well as widespread use of effective medication. Yet, the core issue of asthma control was not accomplished. It is possible that the high prevalence of uncontrolled asthma is partly related to poor adherence. Our results therefore indicate a discrepancy of doctors’ goals and patient perspectives and lack of adequate communication and partnership between the healthcare provider and the patients.

A strength of our study is that it is about asthma patients as seen in real life in the Swedish health care system, in both primary and secondary care. A weakness is that spirometry data were only available for a subset of the sample. As lung function was not recorded in all patients, some of the patients, such as smokers, might have been misdiagnosed with asthma instead of COPD. We also lack information on several potential risk factors such as occupation and physical activity and lack of adherence. It is also possible that prevalence of controlled asthma may have improved since there have been structural changes in the Swedish health system since 2005 with an increase in the number of trained asthma/COPD nurses and responsible asthma/COPD general practitioners (unpublished observations).

In conclusion, this population-based study demonstrates that a large proportion of patients with asthma do not achieve adequate control of their daily symptoms despite appropriate treatment. Furthermore, uncontrolled asthma may have a great impact on patient quality of life. Risk factors for poor asthma control were female sex, older age, low educational level, and smoking, and these groups should therefore be given increased attention in asthma management.
